# The Second Intron Is Essential for the Transcriptional Control of the *Arabidopsis thaliana GLABRA3* Gene in Leaves

**DOI:** 10.3389/fpls.2017.01382

**Published:** 2017-08-08

**Authors:** Alexandra Friede, Bipei Zhang, Stefanie Herberth, Martina Pesch, Andrea Schrader, Martin Hülskamp

**Affiliations:** Botanical Institute, Cologne Biocenter, Cologne University Cologne, Germany

**Keywords:** Arabidopsis, trichomes, patterning, *GLABRA3*, transcriptional regulation

## Abstract

The *GLABRA3* gene is a major regulator of trichome patterning in *Arabidopsis thaliana.* The regulatory regions important for the trichome-specific expression of *GL3* have not been characterized yet. In this study, we used a combination of marker and rescue constructs to determine the relevant promoter regions. We demonstrate that a 1 kb 5′ region combined with the second intron is sufficient to rescue the trichome mutant phenotype of *gl3 egl3* mutants. Swap experiments of the second intron suggest that it is not sufficient to generally enhance the expression level of *GL3*. This implies that the second intron contains regulatory regions for the temporal and spatial regulation of *GL3*. The corresponding GUS-marker constructs revealed trichome-specific expression in young trichomes.

## Introduction

*Arabidopsis* trichomes are single epidermal cells that develop on the surfaces of most aerial organs. Trichomes are regularly distributed on rosette leaves, cauline leaves, sepals and the stem without any obvious reference to other morphological structures (Hulskamp et al., 1994; Hulskamp, 2004; Balkunde et al., 2010). The distribution of trichomes is regulated by gene regulatory network containing genes promoting or inhibiting trichome fate. The positive regulators include the WD40 factor *TRANSPARENT TESTA GLABRA1* (*TTG1*) (Koornneef, 1981; Galway et al., 1994; Walker et al., 1999), the bHLH factors GLABRA3 (Koornneef et al., 1982; Payne et al., 2000; Bernhardt et al., 2003) and the redundantly acting ENHANCER OF GLABRA3 (EGL3) (Bernhardt et al., 2003), and the R2R3MYB factors GLABRA1 (GL1, trichome system) (Oppenheimer et al., 1991) and MYB23 (Kirik et al., 2001, 2005). In addition, several redundantly acting negative regulators, the R3MYB proteins, mediate cell–cell communication by moving between cells (Wada et al., 1997; Schellmann et al., 2002; Kirik et al., 2004a,b; Kurata et al., 2005; Digiuni et al., 2008; Tominaga et al., 2008; Wang et al., 2008; Wester et al., 2009). The trichome promoting genes and the negative regulators are engaged in a regulatory feed back loop that is described in the activator-inhibitor model (Hulskamp, 2004; Pesch and Hulskamp, 2009): The activators TTG1, GL3/EGL3, and GL1/MYB23 form an activator complex, in which TTG1 and the R2R3MYB protein bind to GL3 or EGL3. This complex activates the R3MYBs that in turn can move into neighboring cells where they repress the activators. In addition a so-called activator-depletion mechanism has been postulated according to which TTG1 is mobile in the epidermis and due to its binding to GL3 protein it is trapped in trichomes and depleted in the neighboring cells (Bouyer et al., 2008; Pesch and Hulskamp, 2009; Balkunde et al., 2011).

For a better understanding of the regulatory network it is important to analyze the transcriptional regulation of the key gene *GL3*. In rosette leaves, *GL3 in situ* hybridization experiments have shown that *GL3* is expressed in developing trichomes. The expression analysis of a 2.5 kb 5′-promoter fragment driving the GUS reporter gene revealed a similar expression pattern (Zhang et al., 2003; Zhao et al., 2008) suggesting that the promoter fragment is sufficient for *GL3* function in leaves. As a 1 kb 5′-promoter fragment can rescue *gl3* mutants it is likely that this 1 kb fragment contains all regulatory sequences essential for trichome patterning (Bernhardt et al., 2005). Recently, the 2.5 kb 5′-promoter driving the *GL3* cDNA was shown to rescue the trichome and root hair phenotype of *gl3 egl3* double mutants though the rescue was not complete (Zhao et al., 2012).

In this study, we aimed to identify the relevant promoter regions of the *GL3* gene in the context of trichome patterning. We could not confirm that the 2.5 kb 5′-promoter fragment drives GUS expression in trichomes (Zhang et al., 2003). We also show that a 1 kb 5′-promoter region is not sufficient for rescue. As this upstream region was previously shown to rescue the trichome phenotype when combined with the genomic region of GL3 containing introns and the 3′-1 kb downstream region (Bernhardt et al., 2005) we speculated that introns or the 3′ region contains additional regulatory sequences. A detailed analysis of the function of all introns revealed that intron 2 is essential for rescuing the *gl3* mutant trichome phenotype and that it is sufficient for rescue in combination with the 1 kb 5′-promoter fragment. We exchanged the second intron with intron sequences leading to a generally enhanced expression without rescuing the *gl3 egl3* phenotype. This implies that the intron sequences contain regulatory sequences for the temporal and spatial regulation of *GL3* rather than for an un-specific up-regulation of the *GL3* levels. Finally, we show that the relevant promoter sequences mediate a trichome specific expression of the GUS marker gene.

## Materials and Methods

### Molecular Biology

The 35S promoter cassette of the vector pAMPAT-GW (GenBank accession no. AY436765, Pesch et al., 2015) was exchanged with the 898 bp 5′ sequence immediately upstream of the start codon of *GL3* gene using AscI and XhoI [pAMPAT-GW-GL3(5′-1 kb)]. The 1051 bp 3′ fragment was cloned into the PmeI site of pAMPAT-GW-GL3(5′-1 kb) to create the pAMPAT-GW- GL3(5′-1 kb):LR recombination cassette:(3′-1 kb) vector. All genomic fragments of *GL3* were cloned into pDONR201 by BP reactions (Invitrogen). Deletions of single introns within the genomic sequence of *GL3* were introduced by PCR based site directed mutagenesis. The entry clone carrying the *GL3* gene with the second intron was generated using the following strategy: an entry clone carrying the genomic *GL3* was cut with EcoRV and KpnI generating a *GL3* fragment that includes the second and third intron of *GL3.* This fragment was exchanged against the corresponding *GL3* fragment without introns in the entry clone carrying the coding sequence of *GL3*. Thereafter, the third intron was deleted by PCR based site directed mutagenesis. Coding and genomic sequences of *GL3* were introduced into pAMPAT-GW-GL3(5′-1 kb):LR recombination cassette:(3′-1 kb) by LR recombination with the respective entry clones to generate the various intron deletion constructs. Plants were transformed using the floral dip method described previously (Clough and Bent, 1998).

### Plant Materials and Growth Conditions

Plants were grown on soil at 24°C with 16 h of light per day. All *Arabidopsis thaliana* used in this study were of the Columbia (Col-0) ecotype. The *gl3-3* mutant line has been described previously (Jakoby et al., 2008). *egl3-77439* corresponds to the TAIR accession 1008704039.

### Expression Analysis

Total RNA was extracted from 10-day-old true leaves using the RNeasy Mini Kit (Qiagen, Cat No./ID: 74106) and first-strand cDNA was then synthesized from the total RNA (1 μg) using the RevertAid H Minus 1st strand cDNA synthesis (Thermo) as described by manufacturer’s instruction. Real-time polymerase chain reactions (PCR) contained 1 μl of primer mix (10 μM), 1 μl cDNA template (10-fold dilution),10 μl 2 × SYBR Green master PCR mix and 8 μl water to a total of 20 μl. cDNA concentrations in different samples were normalized with reference to *AtAct2*. Gene-specific primers are listed in Supplementary Table S1.

### Morphological and Histochemical Analysis

GUS stainings were essentially done as previously (Sessions et al., 1999; Schroeder et al., 2016). After staining for 16 h at 37°C, tissues were cleared and leaves were inspected by light microscopy and pictures taken using the DISKUS software (Carl H. Hilgers -Technisches Büro, Germany). Trichome numbers were determined on the third and fourth fully expanded leaf of soil-grown seedlings.

## Results

### 5′-Promoter Region of *GL3* Is Not Sufficient for Proper Expression and Rescue

It has been previously reported, that a 2.5 kb 5′-promoter fragment of *GL3* fused to GUS reveals trichome specific expression in leaves and that a fusion to the *GL3* cDNA can rescue the trichome phenotype in *gl3 egl3* double mutants (Zhang et al., 2003; Zhao et al., 2012). In addition, it was shown that a genomic *GL3* fragment including 1kb of the 5′-promoter was sufficient to rescue the *gl3* trichome phenotype (Bernhardt et al., 2005). To test, whether the 1 kb 5′-promoter fragment is sufficient for trichome-specific expression or whether the introns are also important we created a *pGL3(1 kb):GUS* line. We observed ubiquitous GUS expression in young leaves (Supplementary Figures S1A–D). In older leaves, expression levels were close to background (Supplementary Figures S1E–G).

In parallel, we performed rescue experiments by expressing the *GL3* cDNA under the control of the 1 kb 5′-promoter and 1 kb downstream of STOP codon (termed as 3′-1 kb) in the *gl3-3 egl3-77439* double mutant. The *gl3-3* single mutant shows about 50% reduction in trichome number whereas the *egl3-77439* mutant shows a significant reduction of about 10% similar as reported for the *egl3* allele in the L*er* background (**Table 1** and Supplementary Figures S2A,B) (Zhang et al., 2003). The *gl3-3 egl3-77439* double mutant is completely glabrous and one would expect that rescued lines should exhibit the *egl3 77439* mutant phenotype. Among 80 transformed T1 plants we found no rescue. All plants were completely glabrous (Supplementary Figures S2C,D).

**Table 1:** Trichome number of the third and fourth true leaf in *gl3-3 egl3-77349* mutants transformed with different GL3 rescue constructs.

Genotype	Plant number	Leaf number	Average trichome number
*Col-0*	*n* = 20	3	74.1 ± 17.0
		4	91.2 ± 15.4
*egl3-77439*	*n* = 20	3	68.3 ± 5.5
		4	78.0 ± 6.0
*gl3-3*	*n* = 20	3	19.1 ± 10.2
		4	30.6 ± 8.7
*gl3-3 egl3-77439*	*n* = 20	3	0 ± 0
		4	0 ± 0
p*GL3*:*GL3* (genomic) :3′-1 kb	*n* = 33	3	28.1 ± 18.1
		4	55.1 ± 27.4
p*GL3*:*GL3* (genomicΔintron1) :3′-1 kb	*n* = 36	3	18.2 ± 17.7
		4	32.0 ± 25.4
p*GL3*:*GL3* (genomicΔintron2) :3′-1 kb	*n* = 100	3	0 ± 0
		4	0 ± 0
p*GL3*:*GL3* (genomicΔintron3) :3′-1 kb	*n* = 10	3	39.3 ± 18.0
		4	49.4 ± 24.9
p*GL3*:*GL3* (genomicΔintron4) :3′-1 kb	*n* = 20	3	39.9 ± 17.8
		4	51.5 ± 23.0
p*GL3*:*GL3* (genomicΔintron5) :3′-1 kb	*n* = 16	3	26.5 ± 12.4
		4	35.0 ± 10.1
p*GL3*:*GL3* (genomicΔintron6) :3′-1 kb	*n* = 14	3	18.6 ± 9.0
		4	23.9 ± 12.2
p*GL3*:*GL3* (genomic)	*n* = 20	3	59.0 ± 16.6
		4	64.4 ± 14.0

### The Second Intron of *GL3* Is Essential for Rescue

**FIGURE 1:**
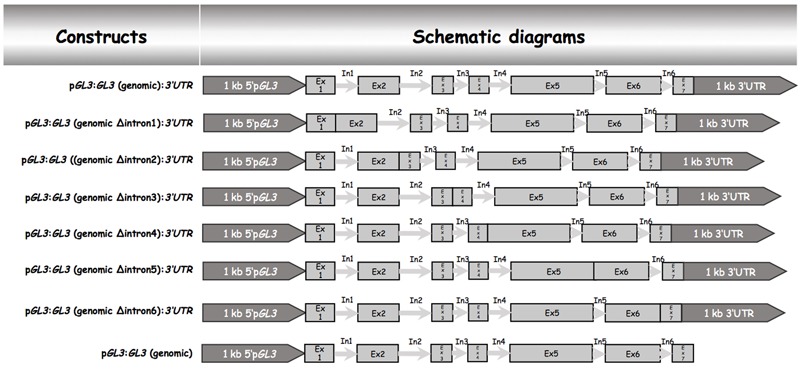
Schematic description genomic *GL3* constructs and intron deletions. All constructs were cloned into the pAMPAT vector backbone. The length of promoter, exon, and intron elements are proportional to the respective sequence length. Ex, exon; In, intron.

Our data indicated that the 5′ promoter region and the 3′-1 kb are not sufficient for rescuing the trichome phenotype of *gl3-3 egl3-77439 double* mutant. We therefore tested the possibility that introns are relevant for the proper regulation of *GL3* during trichome formation. Toward this end, we created a gateway construct containing 1 kb of the 5′ promoter region and 1 kb of the 3′-1 kb [called pGL3:GL3(genomic):3′-1 kb] such that the coding region can be replaced by recombination (**Figure 1**). This construct was used to study the rescue ability in *gl3 egl3* mutants in the T1 generation. As expected, we found a range of rescue phenotypes in the T1 generation. The average rescue efficiency was used as a reference for subsequent analysis (**Table 1**). Next, we created a series of constructs each lacking one of the six introns (**Figure 1**). We found a clear rescue with constructs missing the third, fourth, or fifth intron. The deletion of the first or the sixth intron resulted in a weaker rescue of trichome formation. No rescue was observed in plants carrying the p*GL3*:*GL3* (genomicΔintron 2):3′-1 kb construct (**Table 1**) indicating that the second intron is essential. We therefore focused in the following on the function of the second intron.

To test the relevance of the 3′-1 kb region we studied the rescue in lines harboring the p*GL3*:genomic *GL3* construct (**Figure 1**). These lines showed a rescue of the trichome phenotype. Thus, the 3′-1 kb of *GL3* is not necessary for trichome rescue.

### Analysis of the Function of the Second Intron of *GL3*

**FIGURE 2:**
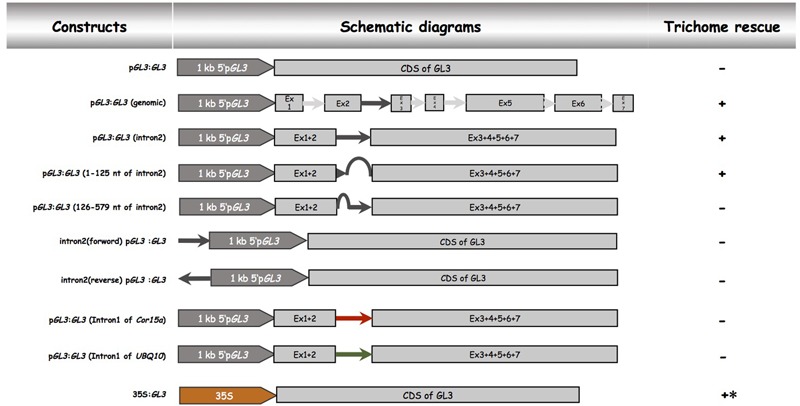
Functional analysis of the second intron of *GL3.* Schematic description of *pGL3* driven genomic *GL3* constructs. All constructs were cloned into the pAMPAT vector backbone. Promoter, exon, and intron elements are proportional to the respective sequence length. + indicates trichome rescue of the *gl3 egl3* double mutant, – indicates no rescue, ^∗^ indicates irregular trichome pattern.

**FIGURE 3:**
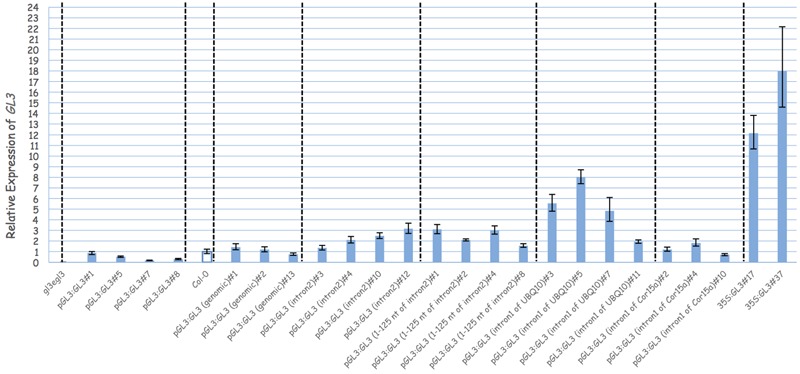
Analysis the function of the second intron of *GL3.* Relative expression of *GL3* in different genotypes. Transcript levels were measured by quantitative real-time PCR and the expression was normalized with reference to the expression of Arabidopsis *Actin2* gene. Error bars are the standard deviations of three technical replicas.

In order to demonstrate that the second intron together with the 1 kb 5′-promoter fragment is sufficient for the transcriptional regulation of *GL3* in the leaf, we expressed the *GL3* cDNA containing the second intron at its original site under the 1 kb 5′-promoter [p*GL3*:*GL3*(Intron 2)] in *gl3 egl3* mutants (**Figure 2**). The majority of T1 lines showed rescue of the trichome phenotype (**Table 2**). In an attempt to map potential relevant regions in the second intron, we compared two deletion constructs missing either the 5′ 125 nt (p*GL3*:*GL3*(Intron 2 delta 3-125) or 454 nt at the 3′end [p*GL3*:*GL3*(Intron 2 delta 126-579)] of the second intron. Only the construct containing the 125 nt at the 5′end rescued the *gl3 egl3* mutant trichome phenotype (**Table 2**) suggesting that this fragment contains all regulatory sequences. In a next step, we assessed whether the position of the intron 2 is important. Toward this end we placed intron 2 in front of the 5′ promoter in both directions (**Figure 2**). Neither construct was able to rescue the *gl3 egl3* trichome mutant phenotype (**Table 2**) indicating that intron 2 does not act as a transcriptional enhancer element. This suggested to us that its position in the transcribed region is important for its function. One well-characterized regulatory mechanism that requires the intron within the transcribed sequence in its correct orientation is intron mediated enhancement (IME) (Rose, 2002; Gallegos and Rose, 2017). To address this possibility, we created two constructs in which the second intron of *GL3* was replaced by introns for which their ability to mediate IME is well characterized (**Figure 2**). The first intron of *UBQ10* resulted in a 2- to 10-fold higher *GL3* expression as compared to wild type Col (**Figure 3**). Insertion of the first intron of the *Cor15a* gene lead to wild-type levels or up to about two-fold increased *GL3* expression (**Figure 3**). By comparison, constructs containing intron 2 or the first 125 nt of intron 2 could enhance *GL3* expression up to three-fold (**Figure 3**). However, neither the *UBQ10* nor the *Cor15a* constructs rescued the trichome phenotype (**Table 2**).

**Table 2:** Rescue of *gl3-3 egl3-77349* trichome phenotype.

Genotype	T1 lines showing trichome rescue (rescued lines/total number of lines)
*gl3-3 egl3-77439*	0/20
p*GL3*:*GL3*	0/20
*Col-0*	20/20
*egl3-77439*	20/20
p*GL3*:*GL3* (genomic)	16/17
p*GL3*:*GL3* (intron 2)	18/20
p*GL3*:*GL3* (1–125 nt of intron 2)	9/10
p*GL3*:*GL3* (126–579 nt of intron 2)	0/15
Intron 2 (forward) p*GL3*:*GL3*	0/16
Intron 2 (reverse) p*GL3*:*GL3*	0/16
p*GL3*:*GL3* (intron 1 of *UBQ10*)	0/11
p*GL3*:*GL3* (intron 1 of *Cor15a10*)	0/20
35S:*GL3*	13/15^∗^

*^∗^Indicates irregular trichome pattern.*

These results suggest that it is not sufficient to merely increase the *GL3* expression by replacing intron 2. It is therefore conceivable that intron 2 is important for the proper regulation of the temporal and spatial expression of *GL3*.

### Expression Analysis of *pGL3: GL3(intron 2)*-GUS

**FIGURE 4:**
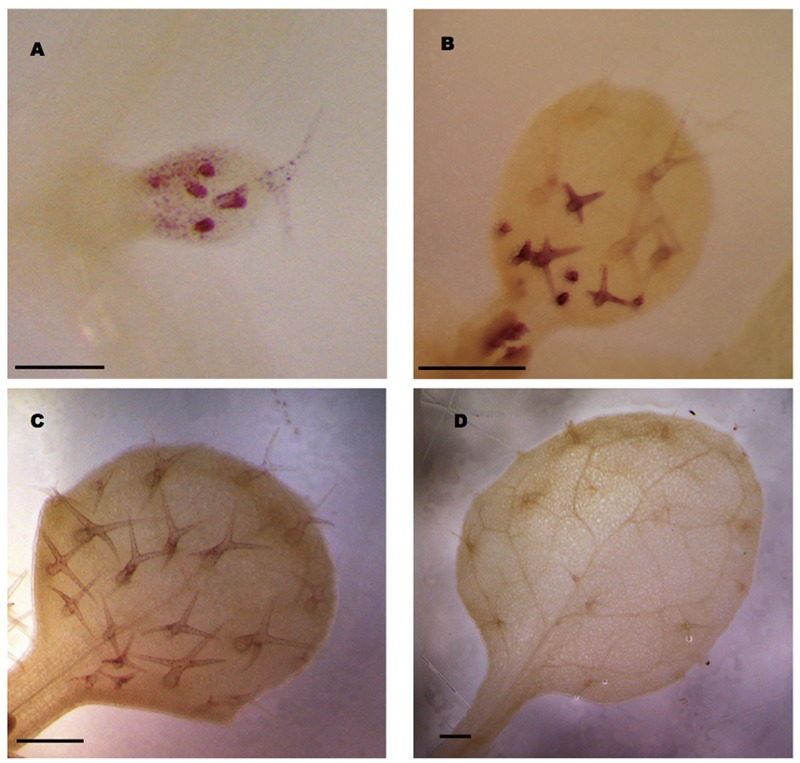
*GL3* expression as revealed by *pGL3:GL3(intron2)-GUS.* Glucuronidase activity was detected in the first or second true leaves using magenta Glc-A as a substrate. **(A)** Incipient leaf with six trichomes at different stages 4 days after germination (DAG). **(B)** Young leaf with young stained trichomes at the leaf base and weakly stained older trichomes at the leaf tip 6 DAG. **(C)** Ten days old mature leaf with weakly stained trichomes. **(D)** Fourteen days old leaf with mature unstained trichomes. Scale bars in **(A–C)** are 100 μm. Scale bar in **(D)** is 200 μm.

Our analysis revealed that a 1 kb promoter fragment combined with intron 2 in the transcribed region of *GL3* is sufficient for complete rescue. To study the expression pattern mediated by this construct we fused the GUS marker gene directly after the second intron. As the signal levels were very low when using X-Gluc as a substrate, we used the more sensitive magenta-Glc-A as a substrate (Schroeder et al., 2016). We detected *GL3* expression in young leaves in all stages of trichome development **Figures 4A,B**). In addition we noted weak expression in epidermal pavement cells in young leaves (**Figure 4A**). In older leaves with young trichome stages at the leaf bases and mature trichomes at the tip of the leaf trichomes exhibited much stronger expression then the mature trichomes (**Figure 4B**). Low levels of *GL3* were maintained during further leaf growth but disappeared in fully mature leaves (**Figures 4C,D**).

## Discussion

In this study, we examined which regions of the *GL3* gene are important for the transcriptional regulation in the context of trichome development. While previous data suggested that the 1 kb 5′ sequences together with 1 kb 3′ sequences might be sufficient for rescuing the trichome phenotype (Zhang et al., 2003; Bernhardt et al., 2005; Zhao et al., 2008) we show that the presence of the second intron is essential and that the insertion of only the second intron in the coding region is sufficient for full rescue in combination with a 1 kb 5′ region. Our data also suggest that intron 2 contains regulatory sequences for the temporal and/or spatial expression of *GL3* as high expression levels mediated by the *UBQ10* intron cannot rescue the trichome mutant phenotype. Consistent with this, the intron 2 of the GL3 gene lies in a region that is hypersensitive to DNase digestion^1^. DNase hypersensitive sites are well-established to indicate regions of active transcriptional elements (Keene et al., 1981; Mcghee et al., 1981) and DNase hypersensitivity of DNA regions in intron 2 support therefore a regulatory role.

This raises the question, how and by which factors intron 2 is regulated to mediate trichome specific expression. One likely scenario would be the regulation by trichome patterning genes, in particular *GL1, GL3*/*EGL3*, and *TTG1*. However, previous studies had shown that the transcriptional regulation of *GL3* does not seem to require any of the known trichome activator genes *GL1, GL3*, or *TTG1*. The total expression level of GL3 is not reduced or absent in *gl1, gl3*, or *ttg1* mutants as judged by RT-PCR experiments (Payne et al., 2000). A possible negative auto-regulation was postulated because overexpression of *GL3* can suppress its own expression (Morohashi et al., 2007). This regulation is likely to be direct as GL3 protein binds to the 5′ region immediately upstream of the transcriptional start site in chromatin-immunoprecipitation experiments (Morohashi et al., 2007). In contrast to the *GL2* and *CPC* promoters the recruitment of GL3 to this promoter region was independent of GL1 (Morohashi et al., 2007). Although these data suggest that *GL3* regulation does not involve *GL1* and *TTG1* this possibility is not ruled out as the exact temporal-spatial expression of *GL3* might be important and not the overall level as measured in PCR experiments. A possible role of transcription factors that are involved in trichome patterning in the regulation of the second intron of GL3 is suggested by several conserved MYB and WRKY (WBOX) binding sites in the second intron of *GL3* (Supplementary Figures S3–S5 and Table S2). Intron 2 and in particular the first 125 nt fragment is generally highly conserved (Supplementary Figure S4) in several Brassicaceae species including *Arabidopsis lyrata, Capsella rubella*, and *Arabis alpina* (Supplementary Table S2 and Figures S3–S5). Strikingly, the relative position of several MYB and WRKY binding sites (WBOXes) is conserved.

Therefore, these binding sites are potentially relevant for the regulation of *GL3* in *Arabidopsis*. For future studies of the temporal and spatial regulation of GL3 it will be helpful that we could map one relevant region down to a fairly small fragment of only 125 nt containing conserved binding sites.

## Author Contributions

AF, BZ, SH, MP, and AS designed, planned and performed the experiments and analyzed the data. MH supervised the project and wrote the manuscript.

## Conflict of Interest Statement

The authors declare that the research was conducted in the absence of any commercial or financial relationships that could be construed as a potential conflict of interest. The reviewer JB and handling Editor declared their shared affiliation, and the handling Editor states that the process met the standards of a fair and objective review.

## Footnotes

1 http://plantdhs.org/plotdhs/TAIR10_Chr5:16530075-16530411
