# Supplementary material for: The Second Intron Is Essential for the Transcriptional Control of the Arabidopsis thaliana GLABRA3 Gene in Leaves
Source: Front Plant Sci. 2017 Aug 8;8:1382. doi: 10.3389/fpls.2017.01382 (PMC5550696; doi:10.3389/fpls.2017.01382)
Supplement: Supplementary file 1 [file Data_Sheet_1.PDF]

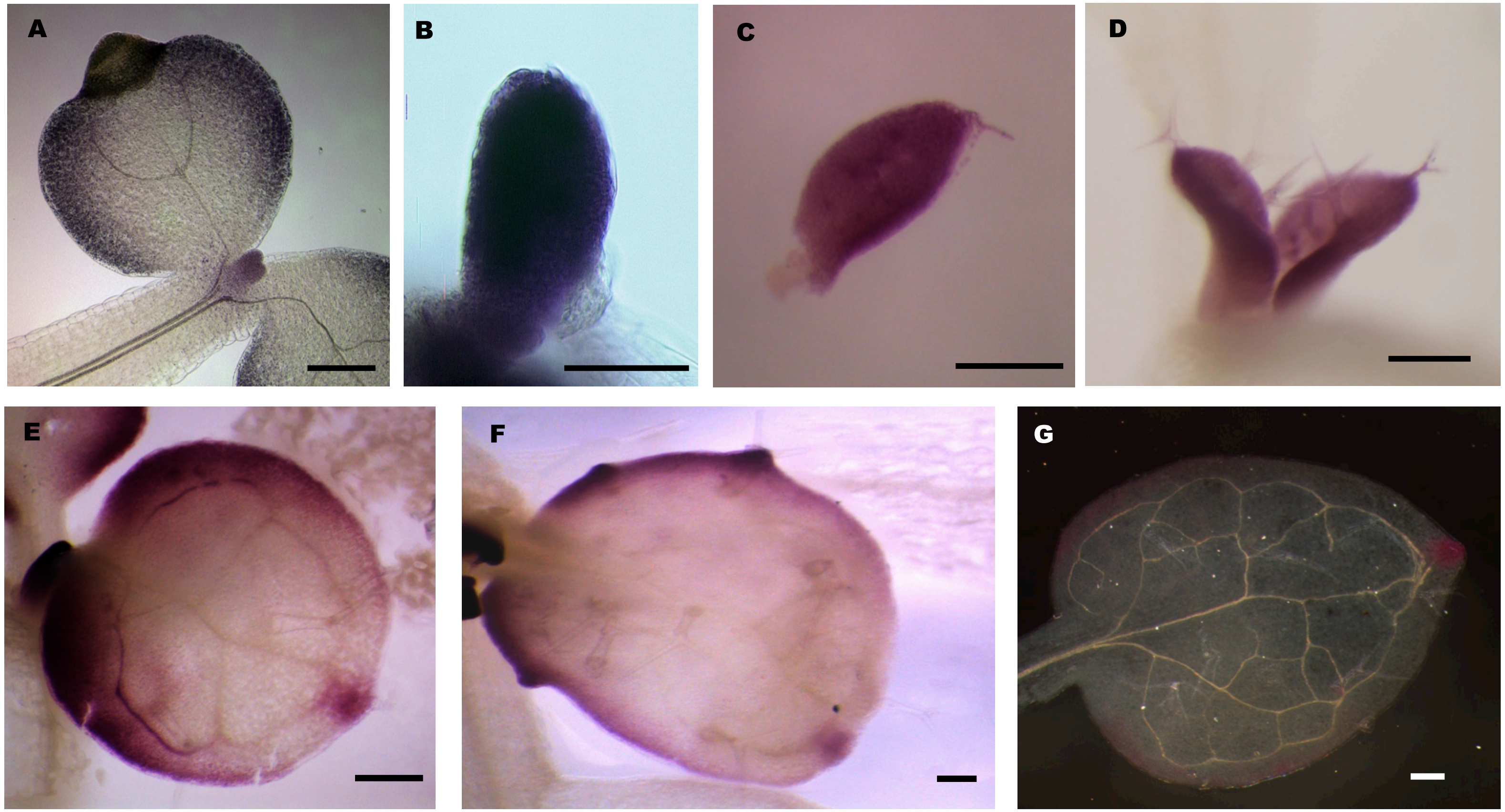

Figure S1: Expression analysis of the 1kb 5' fragment of the *GL3* promoter in leaf.
Expression of the p*GL3(1kb):GUS* reporter fusions in the leaf of different ages seedlings. (A) p*GL3(1kb)*:*GUS* expression pattern in cotyledon; (B-D) p*GL3(1kb)*:*GUS* expression pattern at juvenile stages of true leaf. (E-F) p*GL3(1kb)*:*GUS* expression pattern at mature leaf stages; (G) p*GL3(1kb)*:*GUS* expression pattern in a 12 days old leaf.
Scale bar in A-F=100 µm, Scale bar in G=200 µm.

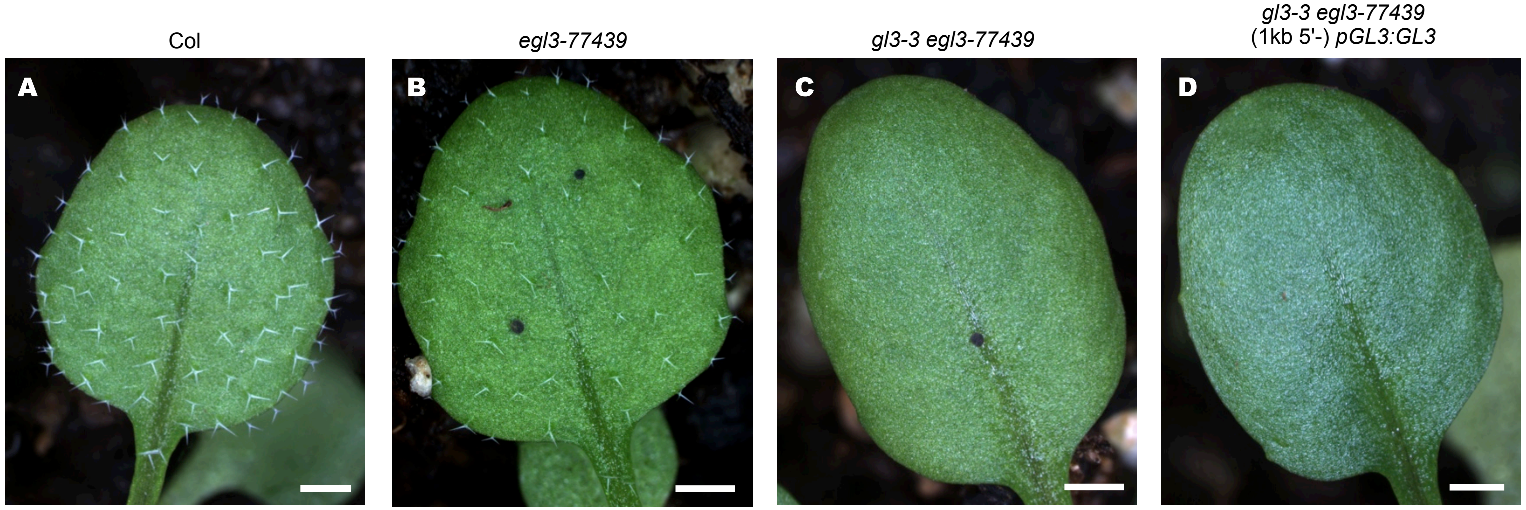

Figure S2: Rescue of *gl3 egl3* double mutants with (1kb 5'-) *pGL3:GL3*.
Third true leaf of a 15 day old wild type Col (A), *egl3-77439* (B), *gl3-3 egl3-77439* (C) and *gl3-3 egl3-77439* (1kb 5'-) *pGL3:GL3.* Bar = 1mm

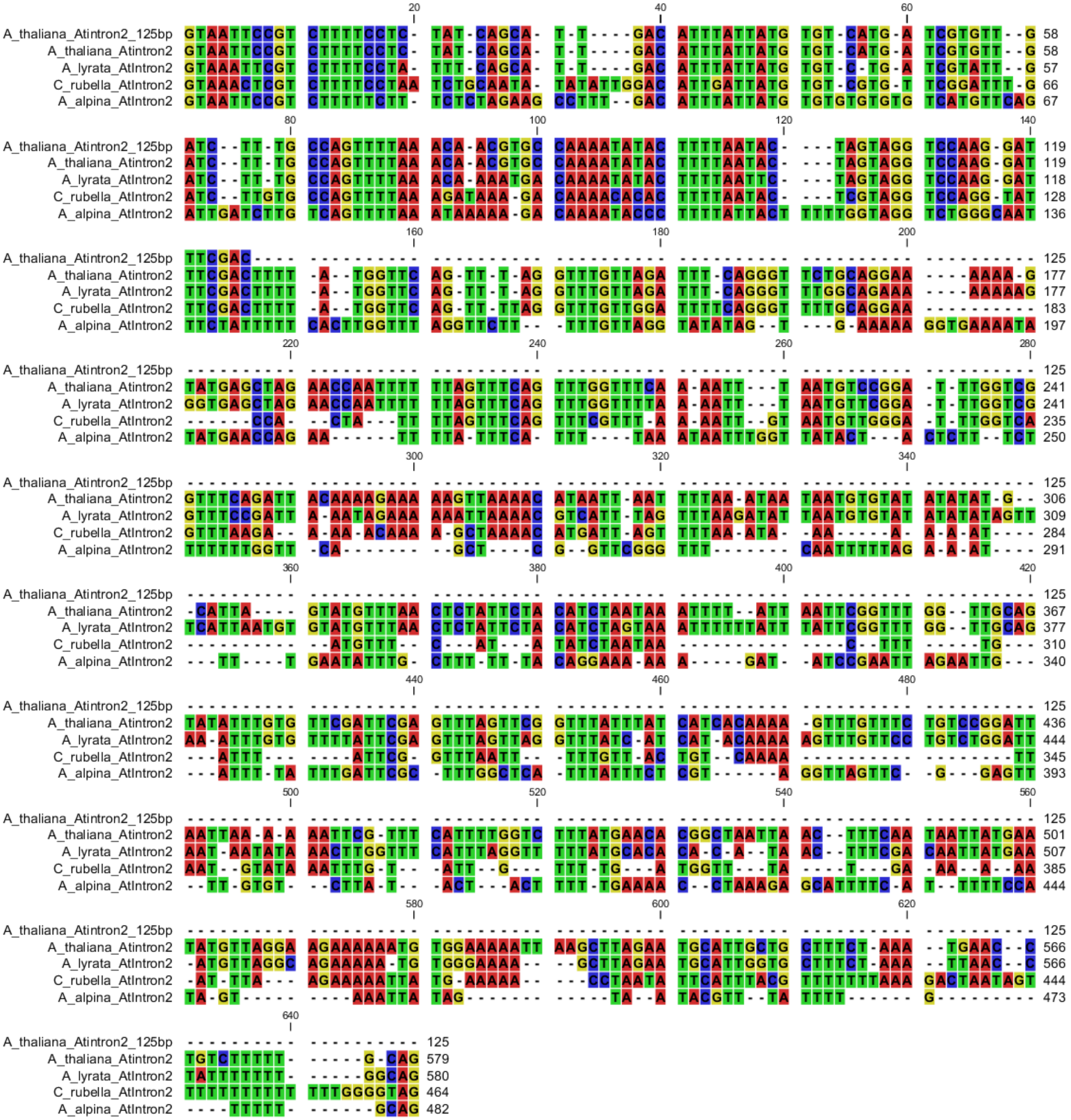
A_thaliana_Atintron2_125bp GTAATTCCGT CTTTTCCTC- TAT-CAGCA- T-T----GAC ATTTATTATG TGT-CATG-A TCGTGTT--G 58
A_thaliana_Atintron2 GTAATTCCGT CTTTTCCTC- TAT-CAGCA- T-T----GAC ATTTATTATG TGT-CATG-A TCGTGTT--G 58
A_lyrata_AtIntron2 GTAAATTCGT CTTTTCCTA- TTT-CAGCA- T-T----GAC ATTTATTATG TGT-C-TG-A TCGTATT--G 57
C_rubella_AtIntron2 GTAAACTCGT CTTTTCCTAA TCTGCAATA- TATATTGGAC ATTGATTATG TGT-CGTG-T TCGGATTT-G 66
A_alpina_AtIntron2 GTAATTCCGT CTTTTTCTT- TCTCTAGAAG CCTTT--GAC ATTTATTATG TGTGTGTGTG TCATGTTCAG 67
A_thaliana_Atintron2_125bp ATC--TT-TG CCAGTTTTAA ACA-ACGTGC CAAAATATAC TTTTAATAC- ---TAGTAGG TCCAAG-GAT 119
A_thaliana_Atintron2 ATC--TT-TG CCAGTTTTAA ACA-ACGTGC CAAAATATAC TTTTAATAC- ---TAGTAGG TCCAAG-GAT 119
A_lyrata_AtIntron2 ATC--TT-TG CCAGTTTTAA ACA-AAATGA CAAAATATAC TTTTAATAC- ---TAGTAGG TCCAAG-GAT 118
C_rubella_AtIntron2 ATC--TTGTG CCAGTTTTAA AGATAAA-GA CAAAACACAC TTTTAATTC- ---TCGTAGG TCCAGG-TAT 128
A_alpina_AtIntron2 ATTGATCTTG TCAGTTTTAA ATAAAAA-GA CAAAATACCC TTTTATTACT TTTTGGTAGG TCTGGGCAAT 136
A_thaliana_Atintron2_125bp TTCGAC---- ---------- ---------- ---------- ---------- ---------- ---------- 125
A_thaliana_Atintron2 TTCGACTTTT -A--TGGTTC AG-TT-T-AG GTTTGTTAGA TTT-CAGGGT TCTGCAGGAA ----AAAA-G 177
A_lyrata_AtIntron2 TTCGACTTTT -A--TGGTTC AG-TT-T-AG GTTTGTTAGA TTT-CAGGGT TTGGCAGAAA ----AAAAAG 177
C_rubella_AtIntron2 TTCGACTTTT -A--TGGTTC AG-TT-TTAG GTTTGTTGGA TTTTCAGGGT TTTGCAGGAA ---------- 183
A_alpina_AtIntron2 TTCTATTTTT CACTTGGTTT AGGTTCTT-- -TTTGTTAGG TATATAG--T ---G-AAAAA GGTGAAAATA 197
A_thaliana_Atintron2_125bp ---------- ---------- ---------- ---------- ---------- ---------- ---------- 125
A_thaliana_Atintron2 TATGAGCTAG AACCAATTTT TTAGTTTCAG TTTGGTTTCA A-AATT---T AATGTCCGGA -T-TTGGTCG 241
A_lyrata_AtIntron2 GGTGAGCTAG AACCAATTTT TTAGTTTCAG TTTGGTTTTA A-AATT---T AATGTTCGGA -T-TTGGTCG 241
C_rubella_AtIntron2 ------CCA- --CTA---TT TTAGTTTCAG TTTCGTTT-A A-AATT--GT AATGTTGGGA -T-TTGGTCA 235
A_alpina_AtIntron2 TATGAACCAG AA------TT TTA-TTTCA- TTT----TAA ATAATTTGGT TATACT---A CTCTT--TCT 250
A_thaliana_Atintron2_125bp ---------- ---------- ---------- ---------- ---------- ---------- ---------- 125
A_thaliana_Atintron2 GTTTCAGATT ACAAAAGAAA AAGTTAAAAC ATAATT-AAT TTTAA-ATAA TAATGTGTAT ATATAT-G-- 306
A_lyrata_AtIntron2 GTTTCCGATT A-AATAGAAA AAATTAAAAC GTCATT-TAG TTTAAGATAT TAATGTGTAT ATATATAGTT 309
C_rubella_AtIntron2 GTTTAAGA-- A-AA-ACAAA A-GCTAAAAC ATGATT-AGT TTTAA-ATA- -AA-----A- A-A-AT---- 284
A_alpina_AtIntron2 TTTTTTGGTT -CA------- --GCT----C G--GTTCGGG TTT------- CAATTTTTAG A-A-AT---- 291
A_thaliana_Atintron2_125bp ---------- ---------- ---------- ---------- ---------- ---------- ---------- 125
A_thaliana_Atintron2 -CATTA---- GTATGTTTAA CTCTATTCTA CATCTAATAA ATTTT--ATT AATTCGGTTT GG--TTGCAG 367
A_lyrata_AtIntron2 TCATTAATGT GTATGTTTAA CTCTATTCTA CATCTAGTAA ATTTTTTATT TATTCGGTTT GG--TTGCAG 377
C_rubella_AtIntron2 ---------- --ATGTTT-- C---AT---A TATCTAATAA ---------- ----C--TTT -----TG--- 310
A_alpina_AtIntron2 ---TT----T GAATATTTG- CTTT-TT-TA CAGGAAA-AA A-----GAT- -ATCCGAATT AGAATTG--- 340
A_thaliana_Atintron2_125bp ---------- ---------- ---------- ---------- ---------- ---------- ---------- 125
A_thaliana_Atintron2 TATATTTGTG TTCGATTCGA GTTTAGTTCG GTTTATTTAT CATCACAAAA -GTTTGTTTC TGTCCGGATT 436
A_lyrata_AtIntron2 AA-ATTTGTG TTTTATTCGA GTTTAGTTAG GTTTATC-AT CAT-ACAAAA AGTTTGTTCC TGTCTGGATT 444
C_rubella_AtIntron2 ---ATTT--- ----ATTCG- GTTTAATT-- -TTTGTT-AC TGT--CAAAA ---------- --------TT 345
A_alpina_AtIntron2 ---ATTT-TA TTTGATTCGC -TTTGGCTCA -TTTATTTCT CGT------A GGTTAGTTC- -G---GAGTT 393
A_thaliana_Atintron2_125bp ---------- ---------- ---------- ---------- ---------- ---------- ---------- 125
A_thaliana_Atintron2 AATTAA-A-A AATTCG-TTT CATTTTGGTC TTTATGAACA CGGCTAATTA AC--TTTCAA TAATTATGAA 501
A_lyrata_AtIntron2 AAT-AATATA AACTTGGTTT CATTTAGGTT TTTATGCACA CA-C-A--TA AC--TTTCGA CAATTATGAA 507
C_rubella_AtIntron2 AAT--GTATA AATTTG-T-- -ATT--G--- TTT-TG---A TGGTT---TA ----T---GA -AA--A--AA 385
A_alpina_AtIntron2 --TT-GTGT- --CTTA-T-- -ACT---ACT TTT-TGAAAA C--CTAAAGA GCATTTTC-A T--TTTTCCA 444
A_thaliana_Atintron2_125bp ---------- ---------- ---------- ---------- ---------- ---------- ---------- 125
A_thaliana_Atintron2 TATGTTAGGA AGAAAAAATG TGGAAAAATT AAGCTTAGAA TGCATTGCTG CTTTCT-AAA --TGAAC--C 566
A_lyrata_AtIntron2 -ATGTTAGGC AGAAAAA-TG TGGGAAAA-- --GCTTAGAA TGCATTGGTG CTTTCT-AAA --TTAAC--C 566
C_rubella_AtIntron2 -AT-TTA--- AGAAAAATTA TG-AAAAA-- ---CCTAATA TTCATTTACG TTTTTTTAAA GACTAATAGT 444
A_alpina_AtIntron2 TA-GT----- ----AAATTA TAG------- -----TA--A TACGTT--TA TTTT------ G--------- 473
A_thaliana_Atintron2_125bp ---------- ---------- 125
A_thaliana_Atintron2 TGTCTTTTT- -----G-CAG 579
A_lyrata_AtIntron2 TATTTTTTT- -----GGCAG 580
C_rubella_AtIntron2 TTTTTTTTTT TTTGGGGTAG 464
A_alpina_AtIntron2 ----TTTTT- ------GCAG 482

Figure S3. Interspecies alignment of Atintron2.

The sequences for the *GL3* gene from *Arabidopsis lyrata* (AL7G36380) and *Capsella rubella* (CRU_007G32850) were extracted from plaza (Dicots PLAZA 3.0, http://bioinformatics.psb.ugent.be/plaza/, Proost et al., 2014) upon blasting for the At*GL3* within their database. The sequence for Aa*GL3* was extracted from genomic sequence data provided by Eva Maria Willig and Korbinian Schneeberger for *A. alpina* ecotype Pajares (Willig et al., 2015). AtIntron2 was identified within these sequences using the protein translation within CLC DNA Workbench (www.clcbio.com). The coding DNA sequence form *A. thaliana* defined the end and begin of the previous and following „exon“, respectively. Please note that it is not given that the splicing occurs in a similar way in other species. The essential part of Intron2 in *A. thaliana* for rescue of the trichome phenotype (AtIntron2_125bp) from *A. thaliana* was additionally aligned to select for this part of AtIntron2 in the other species. Alignment was performed with CLC DNA Workbench and the following settings: gap open cost:2, gap extension cost: 1, end gap cost: as any other, mode: very accurate.

References:

Proost S*, et al.* (2015) PLAZA 3.0: an access point for plant comparative genomics. *Nucleic Acids Res* 43(Database issue):D974-981.

Willing EM*, et al.* (2015) Genome expansion of Arabis alpina linked with retrotransposition and reduced symmetric DNA methylation. *Nat Plants* 1:14023.

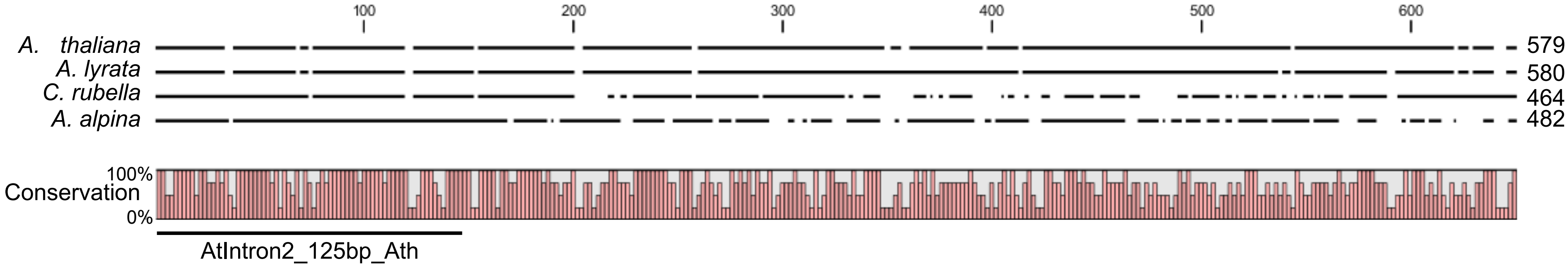

Figure S4. Interspecies conservation of Atintron2.

Alignment of intron2 as shown in Figure S3 extracted from CLC DNA Workbench (www.clcbio.com). The conservation is given as the percentage of identity among the four indicated species. The region of AtIntron2_125bp is marked below. Note the high conservation in the first half of AtIntron2 comprising the 125 nt fragment among the four Brassicaceaes. Next to the alignments, the length of Atintron2 in the respective species is given in base pairs. Numbers above the alignment refer to the respective position within the alignment.

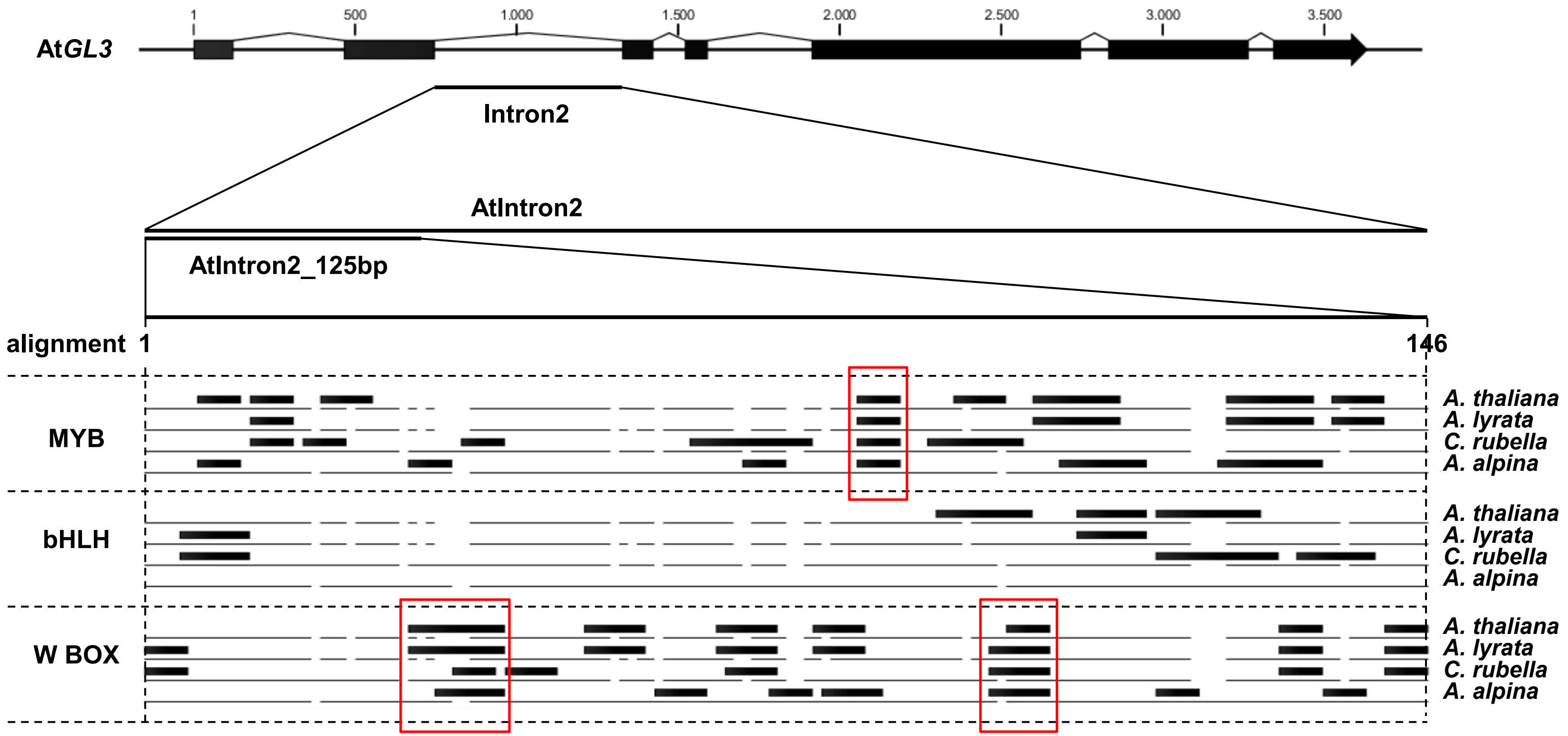

Figure S5: Conservation of MYB, bHLH and WBOX motifs in AtIntron2_125bp.
The exon structure was generated using CLC DNA Workbench (www.clcbio.com). Positions for the At*GL3* exon structure plot are given relative to exon 1 nucleotide 1. The sequence corresponding to AtIntron2_125bp from *A. thaliana* within the other species was determined as described in Figure S3-4. The motifs were identified as described in Table S2 using plantPAN 2.0 (http://plantpan2.itps.ncku.edu.tw/, Chow et al., 2016). The motifs within the AtIntron2_125bp region, as shown here, are placed relative to the position of the whole alignment („alignment“, 146 positions) of this sequence from *A. thaliana*, *A. lyrata*, *C. rubella* and *A. alpina* as shown in Figure 3-4. The alignment is visible in grey below the annotated motifs. Dashed lines serve to separate the different classes of motifs. Red boxes highlight motifs occurring in all species at the same or similar position with the same or similar spacing.

Reference:

Chow CN, *et al.* (2016) PlantPAN 2.0: an update of plant promoter analysis navigator for reconstructing transcriptional regulatory networks in plants. *Nucleic Acids Res* 44(D1):D1154-1160.

Table S1: Primers used in this study

Primer Number	Gene	Sequence (5'–3')
1	GL3-F	CGTCTTCAACATTGGTGAAGGAATG
2	GL3-R_S	TGGTACCAATCTCAACGACTCCTCCAA
3	GL3-R_L	GCGCTTCTTCTCTAAAACCGCATGGT
4	ACT2-F	AGTGGTCGTACAACCGGTATTGT
5	ACT2-R_S	GATGGCATGAGGAAGAGAGAAAC
6	ACT2-R_L	GAAGCAAGAATGGAACCACCGAT
7	5'sgsI-pGL3	GGCGCGCCCGATCACTCAAATAGTAAT
8	3'XhoI-GL3-ex3	CTCGAGGTGGTACCAATCTCAACGACT
